# Intensity modulated radiation therapy and surgery for Management of Retroperitoneal Sarcomas: a single-institution experience

**DOI:** 10.1186/s13014-017-0920-y

**Published:** 2017-12-08

**Authors:** Pippa F. Cosper, Jeffrey Olsen, Todd DeWees, Brian A. Van Tine, William Hawkins, Jeff Michalski, Imran Zoberi

**Affiliations:** 1 Department of Radiation Oncology, Washington University School of Medicine, St. Louis, MO USA; 2 Department of Radiation Oncology, University of Colorado, Aurora, CO USA; 3 Division of Medical Oncology, Department of Medicine, Washington University School of Medicine, St. Louis, MO USA; 4 Department of Surgery, Washington University School of Medicine, St. Louis, MO USA

**Keywords:** Soft tissue sarcoma, Retroperitoneum, Intensity modulated radiation therapy, Surgery, Radiation, Local recurrence, Overall survival

## Abstract

**Background:**

Peri-operative radiation of retroperitoneal sarcomas (RPS) is an important component of multidisciplinary treatment. All retrospective series thus far included patients treated with older radiation therapy (RT) techniques including 2D and 3DRT. Intensity modulated radiation therapy (IMRT) allows for selective dose escalation while sparing adjacent organs. We therefore report the first series of patients with RPS treated solely with IMRT, surgery and chemotherapy. We hypothesized that IMRT would permit safe dose escalation and superior rates of local control (LC) in this high-risk patient population.

**Methods:**

Thirty patients with RPS treated with curative intent between 2006 and 2015 were included in this retrospective study. RT was administered either pre- or post-operatively and IMRT was used in all patients. Statistical comparisons, LC, distant metastasis (DM), and overall survival (OS) were calculated by Kaplan-Meier analysis and univariate Cox regression.

**Results:**

Median follow-up time after completion of RT was 36 months (range 1.4-112). Median tumor size was 14 cm (range 3.6 - 28 cm). The most prevalent histologies were liposarcoma in 10 (33%) patients and leiomyosarcoma in 10 (33%) with 21 patients (70%) having high-grade disease. Twenty-eight (93%) patients had surgical resection with 47% having positive margins. Chemotherapy was administered in 9 (30%) patients. RT was delivered pre-operatively in 11 (37%) patients, and post-operatively in 19 (63%) with 60% of patients receiving a simultaneous integrated boost. Pre-operative median RT dose to the high-risk area was 55 Gy (range, 43–66 Gy) while median post-operative dose was 60.4 Gy (range, 45-66.6 Gy). There was one acute grade 3 and one late grade 3 toxicity and no grade 4 or 5 toxicities. Three year actuarial LC, freedom from DM, and OS rates were 84%, 64%, and 68% respectively. Positive surgical margins were associated with a higher risk of local recurrence (*p* = 0.02) and decreased OS (*p* = 0.04). Pre-operative RT was associated with improved LC (*p* = 0.1) with a 5-year actuarial LC of 100%. Administration of chemotherapy, timing of RT, histology or grade was not predictive of OS.

**Conclusions:**

Patients with RPS treated with peri-operative IMRT at our institution had excellent local control and low incidences of toxicity.

## Background

Retroperitoneal sarcoma (RPS) represents a rare form of heterogeneous soft tissue sarcoma that is challenging to treat due to its deeply seated location and infiltrative nature in the retroperitoneum. Surgical excision is the mainstay of treatment and offers a potential cure when wide resection margins are obtained. However, RPS often presents late and has invaded nearby critical structures making complete excision extremely difficult. It has been shown that incomplete resection and positive microscopic margins predict death from disease and that patients with incomplete resection have equally poor survival as patients with unresectable disease [1]. Given the difficulty in achieving local control, patients with RPS most often die due to local progression, which is in contrast to patients with extremity soft tissue sarcoma who are more likely to die of distant disease [1, 2]. Improving local control is therefore crucial for improving overall survival in these patients.

Perioperative radiotherapy is often used to improve local control, which ranges from 49 to 75% with surgery and radiation therapy (RT) [3–7]. However, achieving adequate dose and target coverage in the retroperitoneum is challenging due to the close proximity of surrounding critical structures such as small bowel, kidneys, liver and spinal cord. RT doses are often limited to 45-50 Gy using older techniques and grade 3 or greater toxicity can approach 40-50% even with these suboptimal doses [8, 9]. Unfortunately, local recurrence rates remain high even if doses of 50-55 Gy can be safely administered with conventional RT [7, 9]. This implies that bowel tolerant doses are not adequate for disease control and that dose intensification is necessary for improved local control. Intra-operative RT (IORT) has been used to deliver a local boost to the margin at risk resulting in improved LC but is also associated with significant toxicity such as an 18% rate of grade 3 gastrointestinal obstruction and moderate to severe peripheral neuropathy [9, 10].

Intensity modulated RT (IMRT) allows for selective dose escalation with simultaneous integrated boosts to high-risk areas while sparing dose to surrounding critical structures. Use of IMRT in the management of RPS has been previously reported [4, 11–13], but all retrospective studies published thus far also included patients treated using older techniques (2D or 3DRT) or with the addition of IORT, which makes it difficult to determine the outcomes with IMRT alone. Small prospective studies have evaluated IMRT but these were in the pre-operative setting only and were limited in their enrollment [14, 15]. The approach at Washington University in St. Louis has been to use IMRT to treat the tumor or tumor bed with bowel tolerant doses and then selectively dose escalate to the margin at risk for recurrence, which is typically the posterior margin. We therefore aimed to report our institutional experience of using IMRT with simultaneous integrated boost either pre- or post-operatively in the management of RPS to determine outcome as well as toxicity. To our knowledge this represents the largest series of RPS treated solely with peri-operative IMRT.

## Methods

We retrospectively identified 30 patients with histologically confirmed soft tissue sarcoma of the retroperitoneum who were definitively treated with IMRT at Washington University in St. Louis between 2006 and 2015. All medical records were reviewed in detail with approval of the Institutional Review Board. All patients underwent complete staging work-up including CT of the chest, abdomen and pelvis. Patients with metastatic disease at diagnosis, desmoid fibromatosis, prior radiation therapy to the tumor, or treatment that was palliative intent were excluded from this analysis.

All patients were evaluated and followed by a multidisciplinary team consisting of surgical, radiation, and medical oncologists. Surgery involved resection of the primary tumor as well as organs that appeared macroscopically involved or were enveloped by the tumor. The decision to resect organs en bloc was made both radiographically and intra-operatively. Organs were not resected if the tumor was thought to have a positive margin in another location. The decision to administer neoadjuvant or adjuvant chemotherapy was dependent upon multiple factors including stage, histology, and grade. Patients who developed metastatic disease after definitive local treatment received salvage chemotherapy per medical oncology discretion. These patients were not considered to have received chemotherapy as a component of their definitive treatment.

**Fig. 1:**
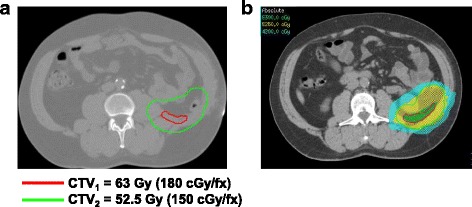
Contours and IMRT treatment plan with a simultaneous integrated boost for a patient with RPS treated with post-operative radiation therapy. **a** Contours on a representative simulation computed tomography scan. Red contour represents the high risk posterior margin that will receive a high dose (CTV1), while the green contour represents CTV2, which provides a lower, bowel tolerant dose to a larger area at risk. **b** IMRT treatment plan with isodose lines. Contours the same as above (red contour = CTV1, green contour = CTV2). The volume receiving 6300 cGy is green, while the lower dose volumes, 5250 cGy and 4200 cGy are yellow and blue respectively

Patients were referred for pre- or post-operative RT based on the discretion of the evaluating surgeon and discussions in a multi-disciplinary conference given concern for extent of anticipated positive margin. In general, this decision was determined by considering factors that influence patient outcomes such as histology, tumor size, tumor depth and anatomic location. If the surgeons did not believe they could achieve an R0 resection, and the morbidity of radiation was thought to be acceptable, then that patient would receive pre-operative RT and all of these patients were considered for simultaneous integrated boost. Patients not referred for pre-operative RT underwent surgery and if histology or margin status provided an indication for RT, then post-operative RT was performed. IMRT was delivered using either sliding window technique or helical tomotherapy. All patients underwent planning CT simulation. For pre-operative RT, the gross tumor volume (GTV) consisted of the entire retroperitoneal sarcoma. In the case of post-operative RT, pre-operative imaging was fused to the simulation CT to create the clinical target volume (CTV), which included the resection bed (posterior margin) including areas with positive surgical margins while avoiding organs that have settled into the post-operative cavity. In all cases, the CTV was expanded beyond the pre-operative GTV by 1.5 cm but respecting hard borders such as bone and unaffected intra-abdominal organs in the case of post-operative RT. Simultaneous integrated boost was delivered to the area at high risk for positive margins following surgical resection. This includes areas where tumor was adjacent to the posterior abdominal wall, intra-abdominal organs, para- and pre-vertebral spaces and major vessels. It is important to note that the “high risk posterior margin” is subjective and input from the surgeon is helpful. In patients who received a simultaneous integrated boost, a high dose was administered to the posterior tumor margin (CTV1) and a second CTV was created (CTV2) which provided a lower, bowel tolerant dose to a larger area at risk for harboring microscopic disease (Fig. 1) as described [16, 17]. The planning target volume (PTV) consisted of a symmetric 0.5 cm margin beyond the CTVs. Daily imaging was used for patient set-up. Organs at risk (OAR) including spinal cord, kidneys, and bowel were contoured and classified as avoidance structures. The following dose constraints and prescription details were used: 95% of prescription dose should cover 95% of PTV; Small bowel max point dose <54 Gy, V45 < 150 cm^3^, V55 < 1 cm^3^; Large bowel max point dose <60 Gy, V30 < 200 cm^3^, V35 < 150 cm^3^, V45 < 100 cm^3^; Stomach max point dose <54 Gy; Liver max point dose <50 Gy, mean < 20 Gy; Kidney V20 < 30% (combined kidney dose); Spinal cord Dmax <45 Gy. Local failure (LF) was defined as any disease recurrence located wholly or partially within the radiation field. Distant metastasis (DM) included disease in the lung, bone, or intra-abdominal organs outside of the radiation field. Follow-up times used in the local control (LC), DM and overall survival (OS) analyses were defined starting from the completion of radiation therapy to the date of first occurrence of the outcome of interest.

Patients were monitored for acute and late toxicity using Common Terminology Criteria for Adverse Events (CTCAE) v4.3 with data obtained by a radiation oncologist from electronic hospital records including weekly on treatment visits and all follow-up visits.

### Statistical analysis

The Kaplan-Meier method was used to calculate actuarial rates of overall survival, local control and distant metastasis. The impact of clinical factors and patient characteristics on LC, DM and OS was evaluated using the log-rank test and univariate Cox proportional hazards models. Multivariate analysis was not performed given only one variable was significant on univariate analysis. All levels of significance were two-sided and *p* < 0.05 was considered statistically significant. SAS v9.4 was used for statistical analysis.

## Results

### Patient and tumor characteristics

Patient and tumor characteristics are listed in Table 1. The majority of patients (*n* = 27, 90%) were treated for primary RPS, while only 3 patients (10%) were treated for recurrent RPS that had been previously treated with surgery alone. There were 15 males (50%) and 15 females (50%) with a median age of 58.5 years (range 18-88 years). Median tumor size was 14 cm (range 3.6 - 28 cm). The majority of patients presented with AJCC stage III disease (*n* = 18, 60%), followed by stage II (*n* = 7, 23%) and stage I (*n* = 5, 17%). The most common histologies were liposarcoma (*n* = 10, 33%, which included de-differentiated liposarcoma (n = 5), well differentiated liposarcoma (*n* = 2), liposarcoma NOS (n = 1), pleomorphic liposarcoma (n = 1) and inflammatory liposarcoma (n = 1)), and leiomyosarcoma (n = 10, 33%). Other histologies were analyzed as one group and included 2 undifferentiated pleomorphic sarcoma (UPS), 2 malignant peripheral nerve sheath tumors, 1 angiosarcoma, 1 PNET/Ewings, 1 mesenchymal chondrosarcoma, 2 malignant spindle cell neoplasms, and 1 sarcoma NOS. The majority of patients had high-grade tumors (*n* = 21, 70%), while 4 (13%) had intermediate grade, 4 (13%) had low grade and grade was not able to be determined in 1 (3%) patient.

**Table 1:** Patient and tumor characteristics

Variable	Value (%)
Age (years)	
Median	58.5
Range	18-88
Sex	
Male	15 (50)
Female	15 (50)
Race	
Caucasian	24 (80)
African-American	4 (13)
Asian	2 (7)
Tumor size (cm)	
Median	14
Range	3.6-28
Stage	
IA	1 (3)
IB	4 (13)
IIA	2 (7)
IIB	5 (17)
III	18 (60)
Histology	
Liposarcoma	10 (33)
Leiomyosarcoma	10 (33)
Other	10 (33)
Tumor Grade	
Grade 1	4 (13)
Grade 2	4 (13)
Grade 3	21 (70)
Unknown	1 (3)
Surgical margins	
R0	12 (40)
R1	12 (40)
R2	2 (7)
Unknown	4 (13)
Chemotherapy	
Yes	9 (30)
No	21 (70)

### Treatment

All patients underwent evaluation for resection of primary or recurrent disease. Two patients did not receive surgery due to low chance of obtaining negative surgical margins and likelihood of significant morbidity. Thus 28 patients (93%) underwent surgery as a main component of their treatment. Adjacent organs that showed evidence of invasion were resected en bloc in 17 patients (57%) and vascular resection and reconstruction was performed when necessary. There were no post-operative deaths. Final surgical margins were negative (R0) in 12 patients (40%), microscopically positive (R1) in 12 patients (40%), grossly positive (R2) with residual disease in 2 patients (7%), and could not be determined in 4 patients (13%). Only 3/11 patients (27%) who received pre-operative RT achieved negative margins at the time of surgery.

All patients received photon radiotherapy using IMRT technique though one patient received a mixed photon/proton plan. One patient required surgical placement of a tissue expander prior to RT, which consisted of two saline implants placed in a Vicryl mesh in order to decrease dose to bowel which had settled into the postoperative renal bed. This implant was removed 1 month after RT with no complications. Eighteen (60%) patients received simultaneous integrated boosts to the margin at risk (Table 2). Radiation was administered pre-operatively to 11 patients (37%) and post-operatively to 19 patients (63%). The median dose delivered to the high-risk margin (CTV1) was 55 Gy (range 43.12 – 66 Gy) pre-operatively and 60.4 Gy (range 45 – 66.6 Gy) post-operatively with fractionation ranging from 180 to 220 cGy per fraction. Overall, 40% of patients received greater than 62.5 Gy to the high-risk margin. In those patients receiving a simultaneous integrated boost, the median dose to areas at risk (CTV2) was 50 Gy (range 45-56 Gy) (Table 2).

**Table 2:** Timing, dose, and characteristics of IMRT treatment

Variable	Value (%)
IMRT	
Pre-operative RT	11 (37)
Post-operative RT	19 (63)
Simultaneous integrated boost	18 (60)
Radiation dose to high risk area, PTV1 (Gy)	
Pre-operative	
Median	55
Range	43-66
Post-operative	
Median	60.4
Range	45-66.6
Radiation dose to low risk area, PTV2 (Gy)	
Median	50
Range	45-56

A total of 9 patients (30%) received chemotherapy. Of these patients, 4 patients (44%) received chemotherapy neoadjuvantly (2 patients received epirubicin and ifosfamide and 2 received vincristine, doxorubicin and cyclophosphamide) and 5 patients (56%) were treated with adjuvant chemotherapy (1 received gemcitabine and taxotere while 4 received epirubicin and ifosfamide).

### Survival and patterns of failure

**Fig. 2:**
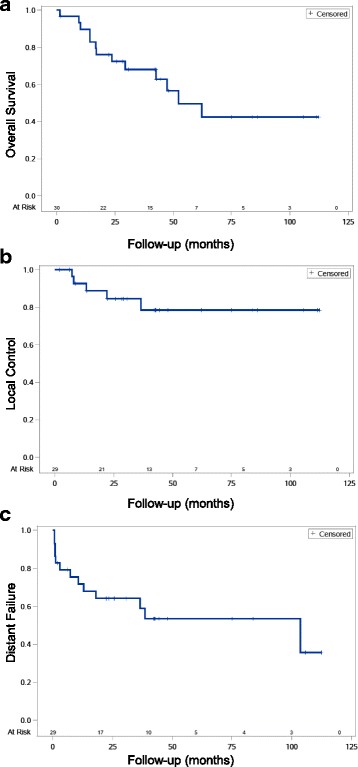
Kaplan-Meier curves for (**a**) overall survival, (**b**) local control, and (**c**) distant metastasis for all patients with RPS treated with IMRT

The median follow-up was 36.3 months (range 1.4 – 112 months). A total of 13 patients (43%) had died at last follow-up and actuarial 3 and 5 year OS was 68% and 50% respectively (Fig. 2a). Timing of radiotherapy, histology, tumor grade and treatment with chemotherapy were not associated with OS (Table 3). Surgical margin status, however, was correlated with OS as patients with negative margins had 3- and 5-year OS of 92% and 78% while those with positive margins (R1) had 3- and 5-year OS of 52% and 35% respectively (*p* = 0.09). R1 resection was significantly associated with OS on univariate cox analysis (*p* = 0.04) though this should be interpreted with caution given the small numbers in this study.

**Table 3:** Univariate log-rank analysis of IMRT timing, surgical margin status, chemotherapy, histology, and tumor grade on OS, LC and DM

	3-year rate (%)	5-year rate (%)	*p* value
Overall Survival			
Radiation therapy			
Pre-operative RT	80	54	0.85
Post-operative RT	62	48	
Surgical Margins			
Negative (R0)	92	78	0.09
Positive (R1)	52	35	
Chemotherapy			
Yes	67	53	0.74
No	69	48	
Histology			
LMS	77	58	0.48
Liposarcoma	67	50	
Other	60	45	
Grade			
Low	86	57	0.58
High	65	50	
Local Control			
Radiation therapy			
Pre-operative RT	100	100	0.10
Post-operative RT	77	69	
Surgical Margins			
Negative (R0)	100	100	0.02
Positive (R1)	79	60	
Chemotherapy			
Yes	65	65	0.16
No	94	85	
Histology			
LMS	100	100	0.06
Liposarcoma	100	75	
Other	58	58	
Grade			
Low	100	100	0.09
High	77	68	
Distant Metastasis			
Radiation therapy			
Pre-operative RT	32	55	0.91
Post-operative RT	38	45	
Surgical Margins			
Negative (R0)	25	34	0.16
Positive (R1)	27	45	
Chemotherapy			
Yes	55	55	0.69
No	26	44	
Histology			
LMS	50	50	0.53
Liposarcoma	11	33	
Other	40	55	
Grade			
Low	25	40	0.54
High	40	49	

**Fig. 3:**
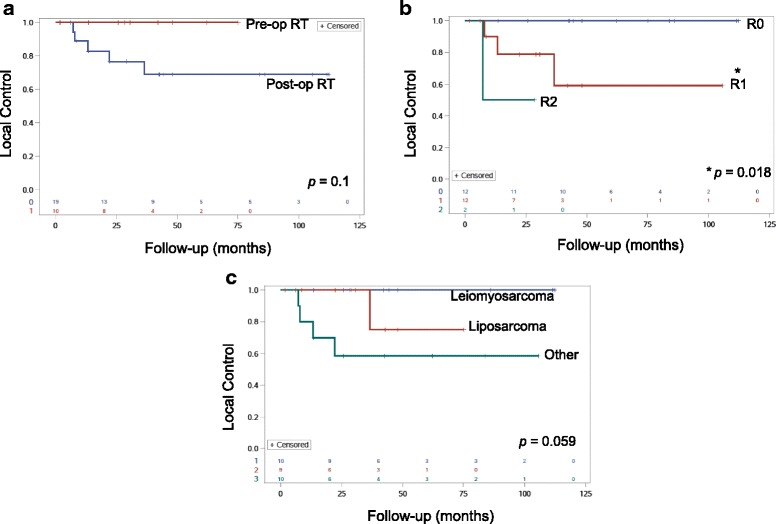
Kaplan-Meier curves for local control for patients with RPS stratified by (**a**) pre-operative (pre-op, red) versus post-operative (post-op, blue) RT, (**b**) surgical margin status, and (**c**) histology. Surgical margin status was significantly associated with local control (*p* = 0.018)

A total of 5 patients (17%) experienced LF and 3- and 5-year actuarial LC was 84% and 79% respectively (Fig. 2b). All patients who failed locally received RT post-operatively. LC in this group was 77% and 69% at 3 and 5 years while patients who received pre-operative RT had 100% LC at 3 and 5 years (*p* = 0.1, Fig. 3a). LF occurred despite a high dose to the high-risk margin. Three of five patients with LF received 63-66.6 Gy, while the other two patients received 50 and 45 Gy. Surgical margin status was a statistically significant predictor of LC. Patients who received an R0 resection had 100% LC at 3 and 5 years whereas patients receiving an R1 resection had 79% and 60% LC at 3 and 5 years respectively (*p* = 0.018, Fig. 3b). Both patients with an R2 resection failed locally and died within 2 years of treatment. Patients with low grade (grade 1-2) tumors had 100% LC at 3 and 5 years, while patients with high grade tumors had 77% and 68% LC at 3 and 5 years respectively (*p* = 0.09**).** Tumor histology was also associated with local control as patients with leiomyosarcoma had 100% LC at both 3 and 5 years, while patients with liposarcoma had 100% and 75% LC at 3 and 5 years respectively. Patients with other histologies did worse overall and had a 58% LC at both 3 and 5 years (*p* = 0.059, Fig. 3c). No clinical variables were significantly correlated with LC on univariate analysis. Importantly, all patients that failed locally had died at last follow-up.

Overall, 13 patients (43%) failed distantly. The actuarial rate of DM at 3 and 5 years was 36% and 47% respectively (Fig. 2c). The most common first site of DM was lung in 46% of patients followed by liver (23%) and abdominal sites outside of the radiation field (23%). Sites of DM after the first metastatic occurrence included lung in 10/13 (77%) patients, liver in 5/13 (38%) patients, and other intra-abdominal sites in 4/13 (31%) patients. Of note all patients that developed liver metastasis at any time had intraparenchymal metastasis indicating hematologic spread. Overall rate of DM did not differ between patients receiving RT pre- or post-operatively and was 32% and 38% at 3 years and 55% and 45% at 5 years respectively however, all patients who developed metastasis to intra-abdominal sites outside of the radiation field received post-operative RT indicating that pre-operative RT may reduce the risk of intra-abdominal spread. Extent of surgical resection, grade, histology, and treatment with chemotherapy did not affect the incidence of DM (Table 3). No clinical variables were significantly correlated with DM on univariate analysis. DM was not associated with LR as 9/13 patients (69%) developed DM without LR while only 4/13 patients (31%) developed both LR and DM. Overall, 77% of patients who developed DM had died at last follow-up.

### Toxicity

Only two patients (7%) developed Grade 3 toxicities. One patient who received pre-operative RT developed acute grade 3 nausea/vomiting requiring intravenous hydration and her treatment was discontinued after 43 Gy (out of a planned 49 Gy). Another patient developed a duodenal stricture 6 months after post-operative RT (50.4 Gy), which required dilation. There were no grade 4 or 5 toxicities.

## Discussion

Treatment of RPS remains extremely challenging and long-term overall survival remains poor despite advances in surgical and radiotherapy technique. Local recurrence has historically been the predominant failure pattern in patients with RPS and accounts for the majority of deaths [1, 7, 18]. Conversely, our series with IMRT revealed that most patients died due to DM with few LF, however all patients with a LF died of disease. This may be explained by the ability to selectively dose escalate to high-risk margins with IMRT, which was not possible using older radiotherapy techniques. Similar results were found in a randomized trial comparing EBRT with or without an IORT boost where IORT improved LC but had higher rates of DM [9]. Though our data reveals that IMRT is able to achieve excellent LC, it did not translate into improved OS compared to other published series. This is likely due to the incidence of DM, which is slightly higher than previously reported experiences [4, 6]. Perhaps the improved LC led to increased short-term survival which allowed time for development of DM. Interestingly, the majority of patients with DM did not have a LR which implies the need for better and perhaps more aggressive systemic therapy.

IMRT allows for selective dose escalation to high-risk margins while allowing for maximal sparing of OAR and has been employed pre-operatively in a small series with excellent LC [15] and is currently being evaluated in a clinical trial (NCT01841047). Using a simultaneous integrated boost, we were able to achieve even higher doses to the margin at risk since over 40% of patients received greater than 62.5 Gy to CTV1. This resulted in excellent 5-year local control of 79%, which is superior to most other published series. However, three of the five patients who failed locally received 63, 66, and 66.6 Gy post-operatively. Failure therefore occurred despite a high dose to the high-risk margin, implying inherent radioresistant biology of some sarcomas, which may require further dose escalation, or tumor infiltration beyond the target. However all of these patients had high-grade disease, two had microscopically positive margins and one had gross residual disease, reinforcing the importance of a complete resection. Indeed, we revealed that surgical margin status was the only factor significantly associated with both LC and OS on log-rank test and univariate analysis respectively as has been shown in numerous prior studies [3, 4, 7]. Given that LR occurred in patients with positive margins despite high doses of radiation, it appears that high doses of RT cannot compensate for inadequate surgery. Proton therapy also allows for local dose escalation with minimal toxicity and a phase I dose escalation trial in the pre-operative setting has shown promising results [19]. A phase I/II dose escalation trial comparing pre-operative IMRT and intensity modulated proton therapy (IMPT) is currently accruing (NCT01659203) and will provide insight into determining the safest and most efficacious radiation modality.

Though higher doses are historically associated with improved LC [5, 18], they come at the cost of greater toxicity. Historically, conventional EBRT is associated with a high rate of grade 3 or greater gastrointestinal toxicity [3, 9, 13] though this rate has been decreasing with more advanced technology [4, 13]. Intra-operative RT (IORT) and brachytherapy have been used to provide a local boost to the high-risk margin while sparing surrounding organs though is technically difficult and is also associated with significant side effects including debilitating neuropathy which ranges from 6 to 33% and an 18-30% rate of severe, grade 3-4 GI side effects [9, 10, 20]. IMRT allows the dose to conform more precisely to the three-dimensional shape of the tumor while minimizing dose to the surrounding critical structures allowing local dose escalation with fewer side effects. Our results compare favorably to the LC rates achieved with IORT but with less toxicity.

There is retrospective evidence that IMRT provides a dosimetric advantage and is associated with significantly less toxicity than 3DRT [13, 21]. Here we report a grade 3 toxicity rate of 7% using IMRT. Interestingly, the two patients with grade 3 toxicities received low doses (43 and 50.4 Gy) of radiation implying that perhaps their biology rather than the dose was involved in the development of toxicity. No patients who received greater than 50.4 Gy experienced a grade 3 toxicity implying we were able to adequately spare OAR using our technique with IMRT and simultaneous integrated boost.

There have been no randomized prospective trials comparing pre- to post-operative RT but pre-operative radiation has become the modality of choice for large, high grade RPS as this increases the likelihood of achieving a resection with negative margins [22] and is associated with less toxicity [3, 4]. Our results reveal that patients who received pre-operative RT had 100% LC at both 3 and 5 years which supports the use of IMRT in the pre-operative setting. Interestingly, patients receiving pre-operative RT had a low rate of negative surgical margins which is likely explained by the patient population that was selected for this modality in the first place; namely patients with less favorable tumor characteristics with a low chance of obtaining negative surgical margins. There was no difference in toxicity between patients who received pre- or post-operative RT though our toxicity rate was low which limits interpretation of this result. Post-operative RT has historically been associated with higher acute and long-term morbidity but our findings using IMRT do not support this as only 1/19 patients (5%) receiving post-operative RT experienced a grade 3 toxicity. Though pre-operative RT is currently the modality of choice based upon several retrospective studies, we show here that using IMRT in the post-operative setting can achieve good LC with minimal toxicity.

There are several limitations to this study that are inherent in most RPS studies. Our small sample size is due not only to the rarity of this disease, but also the relatively recent adoption of IMRT technique. Given the small sample size and low number of events, statistical significance must be interpreted with caution and results may be considered as positive associations rather than statistical conclusions. Additional challenges with this dataset are multiple histologies, variability in grade and tumor size, and inclusion of patients with recurrent disease. Of the three patients that were treated for recurrent disease, two were treated with RT post-operatively, both of which died of disease and one patient was treated pre-operatively and had no evidence of disease at last follow-up. The small number of patients and events in this study also limited univariate and multivariate analysis. This study is retrospective in nature, and there is some bias regarding the patients selected for RT and the timing of RT with surgery. However, there is no inherent treatment bias with regard to radiation technique as all patients treated with RT after the year 2006 were treated with IMRT. A prospective, randomized trial is required to fully investigate the proposed benefit of IMRT yet this is unlikely to occur given the known dosimetric advantages and decreased toxicity associated with IMRT over 3DRT.

In conclusion, though there is no randomized evidence to support the use of peri-operative RT as a component of definitive treatment of RPS there are several large retrospective series that reveal a benefit with RT. One of the largest multi-institutional retrospective series including over 1000 patients with RPS found that peri-operative administration of RT was statistically significantly associated with improved LC [6], and recent analysis of the NCDB and SEER databases revealed that peri-operative radiotherapy was associated with a significant increase in OS compared to patients who were treated with surgery alone [23, 24]. We eagerly await the results of the phase 3 randomized trial (STRASS, NCT01344018) comparing surgery alone with pre-operative RT and surgery as this will provide an answer as to whether RT improves recurrence-free survival.

## Conclusions

Here we report for the first time a cohort of patients with RPS treated solely with IMRT, which resulted in excellent LC and low rates of toxicity compared to older series, which all included patients treated with standard 2D, 3DRT or IORT. However, positive surgical margins and high-grade disease continue to be poor prognostic factors regardless of the RT modality. With continued improvements in radiotherapy technology the therapeutic ratio will hopefully continue to improve. Unfortunately, OS of patients with RPS remains poor, which emphasizes the need for improved local and systemic therapy.

## Abbreviations

2D: 2-dimensional
3D: 3-dimensional
CTCAE: Common Terminology Criteria for Adverse Events
CTV: Clinical target volume
DM: Distant metastasis
EBRT: External beam radiation therapy
GTV: Gross tumor volume
IMPT: Intensity modulated proton therapy
IMRT: Intensity modulated radiation therapy
IORT: Intra-operative radiation therapy
LC: Local control
LF: Local failure
NCDB: National Cancer Database
NOS: Not otherwise specified
OAR: Organs at risk
OS: Overall survival
PNET: Primitive neuroectodermal tumor
PTV: Planning target volume
RPS: Retroperitoneal sarcoma
RT: Radiation therapy
SEER: Surveillance, Epidemiology, and End Results
UPS: Undifferentiated pleomorphic sarcoma
